# Parasitism rate differs between herbivore generations in the univoltine, but not bivoltine, range

**DOI:** 10.1371/journal.pone.0294275

**Published:** 2023-11-27

**Authors:** Álvaro Gaytán, Igor Drobyshev, Tatiana Klisho, Karl Gotthard, Ayco J. M. Tack

**Affiliations:** 1 Instituto de Recursos Naturales y Agrobiología de Sevilla, Spanish National Research Council (IRNAS-CSIC), Seville, Spain; 2 Department of Ecology, Environment and Plant Sciences, Stockholm University, Stockholm, Sweden; 3 Bolin Center for Climate Research, Stockholm University, Stockholm, Sweden; 4 Southern Swedish Forest Centre, Swedish University of Agricultural Sciences, Alnarp, Sweden; 5 Department of Zoology, Stockholm University, Stockholm, Sweden; Government College University Faisalabad, PAKISTAN

## Abstract

With climate change, plant-feeding insects increase their number of annual generations (voltinism). However, to what degree the emergence of a new herbivore generation affects the parasitism rate has not been explored. We performed a field experiment to test whether the parasitism rate differs between the first and the second generations of a specialist leaf miner (*Tischeria ekebladella*), both in the naturally univoltine and bivoltine parts of the leaf miner’s distribution. We found an interactive effect between herbivore generation and geographical range on the parasitism rate. The parasitism rate was higher in the first compared to the second host generation in the part of the range that is naturally univoltine, whereas it did not differ between generations in the bivoltine range. Our experiment highlights that shifts in herbivore voltinism might release top-down control, with potential consequences for natural and applied systems.

## Introduction

With climate change, insects not only advance their phenology but also frequently increase their number of annual generations (voltinism) [1, 2]. Such changes in insect voltinism can create a temporal mismatch between host insects and their natural enemies [3]. If natural enemies are not able to track changes in the voltinism of their host, additional herbivore generations might enter enemy-free space, potentially resulting in higher infestation levels and increased plant damage in natural and agricultural systems [4]. While parasitoids are major natural enemies of insect herbivores, the impact of shifts in the voltinism for host-parasitoid interactions remains largely unexplored [5].

Herbivores have frequently evolved traits that allow them to enter a point in space or time where the natural enemy is not present or active, a concept commonly referred to as enemy-free space [4]. Likewise, herbivores have often dispersed to locations where the natural enemies are not present, for example, through rare long-distance dispersal events [6–8]. When the host and natural enemies respond differently to the environment, enemy-free space might also appear due to changes in the environment [6, 7]. Currently, several herbivore species are increasing their number of annual generations in response to climate change [1, 2, 9]. As natural enemies might not switch voltinism at the same time as their host, the herbivores might (at least temporarily) enter enemy-free space [1, 2, 9]. The occurrence of such enemy-free space will depend on the level of specialization of the natural enemy community, as well as the mechanisms behind host-natural enemy synchrony [5–7, 10]. For example, if the herbivore is attacked by generalist natural enemies, the herbivore might be readily attacked by its current set of natural enemies also during the new generation: While the host might not have been previously present during a certain time period, the generalist natural enemy might already have been feeding on other hosts during that time period [5, 10, 11]. On the other hand, if the herbivore is attacked by specialist insects whose voltinism is not physiologically linked to the herbivore, enemy-free space is to be expected.

In this study, we performed a field experiment to simulate the production of a novel second generation in a naturally univoltine area to test if this additional generation would encounter enemy-free space. More specifically, we targeted a leaf miner with a specialized parasitoid community to test the hypothesis that the parasitism rate would be lower in the second compared to the first generation within the part of the range that is normally univoltine, whereas we would not expect such differences in the parasitism rate between the first and second generations in areas where the herbivore is naturally bivoltine.

## Methods

### Study system

The pedunculate oak *Quercus robur* L. (Fagaceae) is a widely distributed foundation tree in Europe that reaches the northern part of its distribution in Fennoscandia [12, 13]. In Sweden, oaks budburst takes place during late May, and leaf senescence starts between late August and early September [14]. The leaf miner *Tischeria ekebladella* Bjerk. (Tischeriidae) is a specialist moth species on oaks distributed across Europe. *T*. *ekebladella* is univoltine in the northernmost part of its distributional range in central Sweden and bivoltine in southern Sweden [2, 15, 16]. The natural parasitoid community of *T*. *ekebladella* is mainly characterized by specialist parasitoids belonging to the hymenopteran family Eulophidae (e.g. *Chrysocharis* spp. and *Pnigalio* spp.), and to a lesser extent, by the Braconidae (e.g. *Colastes braconius*) and Ichneumonidae (e.g. *Scambus inanis*) families [9–11]. While there is some overlap between the identities of the parasitoids that attack *T*. *ekebladella* and other leaf miner species, the degree of quantitative overlap is rather low. Previous studies have found no evidence for apparent competition or facilitation [17, 18].

### Field experiment

**Fig 1:**
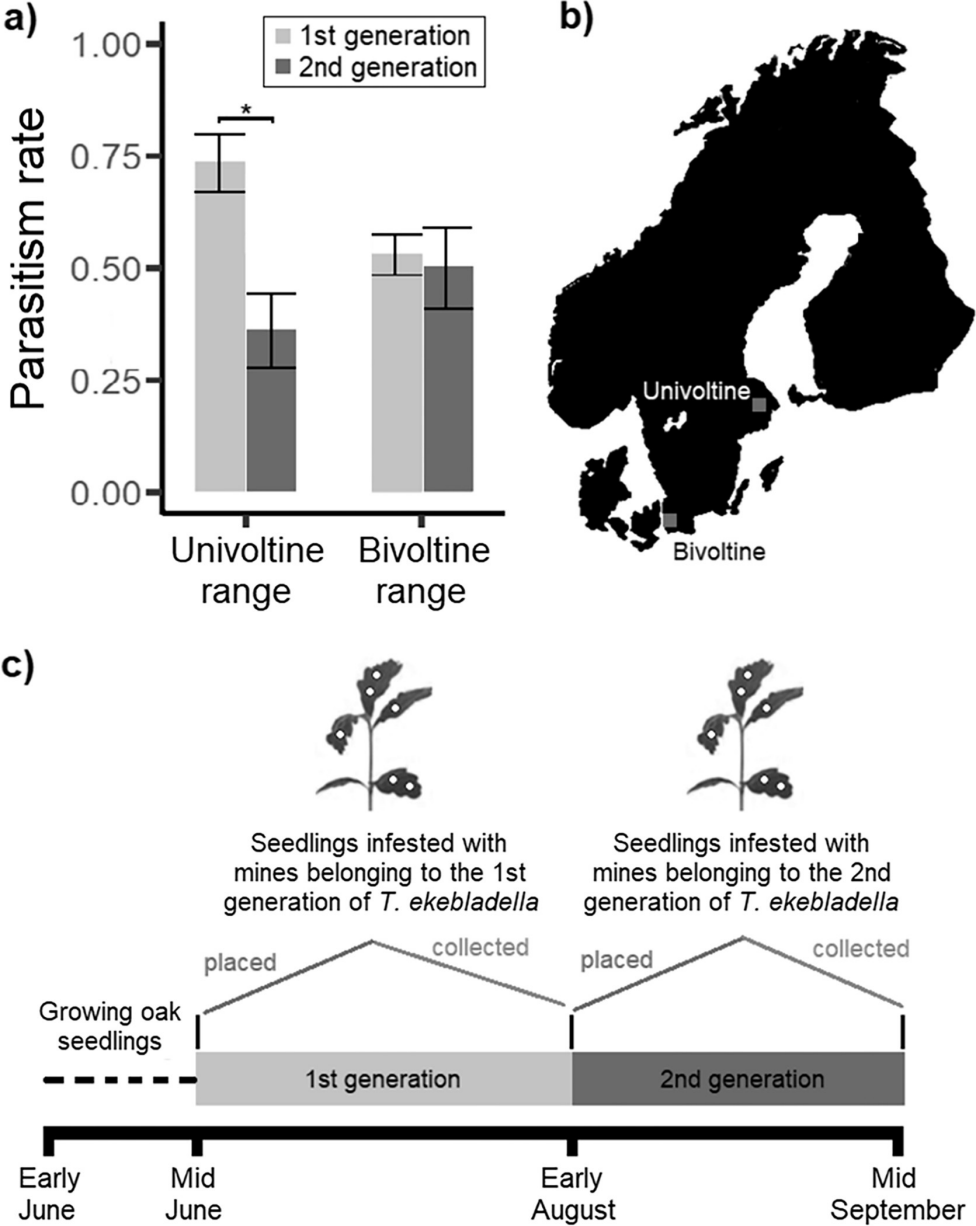
Overview of the study location, experimental timeline and key findings. Panel **a)** shows differences in the parasitism rates between the first and second generations of the leaf miner *Tischeria ekebladella* in the univoltine and bivoltine ranges, respectively. Black bars represent standard errors, and an asterisk represents a statistically significant difference (P < 0.05) between generations within a given range. Panel **b)** shows the location of the study site in the univoltine (Stockholm, Sweden) and bivoltine range (Alnarp, Sweden) of the specialist leaf miner *Tischeria ekebladella*. Panel **c)** depicts the experimental timeline, illustrating when we placed seedlings with leaf mines of the first and second generations in the field and when we collected those seedlings for assessment of parasitism rate.

We selected the Royal National City Park in Stockholm (central Sweden) as an example of the univoltine range of *T*. *ekebladella* (Latitude: 59.366286, Longitude: 18.046509) and the university campus of the Swedish University of Agricultural Sciences in Alnarp (southern Sweden) as an example of the bivoltine range of *T*. *ekebladella* (Latitude: 55.653329, Longitude: 13.076023), as based on observations registered in the database Artportalen and previous studies on voltinism, as shown in Fig 1 [2, 19]. Since we established our experiment at our respective institutions, and we worked with a native tree and herbivore species, no permits were required. In early June 2020, we infested a set of 80 oak seedlings in a growth chamber at 23°C and 20 h of light using adults of *T*. *ekebladella* reared from mines that were collected in the area of Stockholm in October 2019. Two weeks after oak budburst in each site (approximately mid-June 2020), we placed 40 seedlings infested with leaf mines smaller than 2 mm in each site (approximately one week after *T*. *ekebladella* females laid eggs), with 4 ± 1 (mean ± SD) mines per plant (Fig 1). Within each location, plants were distributed randomly across a 30 × 20 m area. In the beginning of August, when the second generation of *T*. *ekebladella* naturally starts in the bivoltine range [19], we repeated the described process of infestation of seedlings (Fig 1). By substituting the first set of plants with a second one in both sites, we simulated a second generation of *T*. *ekebladella*. The total number of seedlings used in this study was 160 (n = 40 per site and generation of *T*. *ekebladella*) and larvae were exposed to parasitoids during approximately five weeks per generation. Seedlings were watered when needed.

Upon collection of the seedlings (August and September 2020 for the first and second herbivore generations, respectively), the mines were scored as either (i) adult moth emerged, as evident by a large, ~ 1 mm rupture of the mine, (ii) adult parasitoid emerged, as evident by a small (< 1mm) circular rupture of the leaf mine, or (iii) leaf mine with unbroken surface [15, 17]. Leaf mines with unbroken surface were individually reared in pots. If rearing was unsuccessful, mines and larvae were dissected under the microscope to identify traces of parasitism [10, 13, 20, 21].

### Statistical analysis

We modelled the probability of parasitism as a function of host generation, host range and the interaction between host generation and host ranges using a generalized linear mixed effects model with a binomial distribution with a logit link. Since we had multiple mines on the same plant, and sometimes on the same leaf, we included plant and leaf identity as random factors. We then tested for differences in parasitism rate between the first and second generations, separately for the univoltine and bivoltine ranges. To implement the generalized linear mixed effects model, we used the function *glmer* from the *R*-package *lme4* [22, 23]. We assessed model fit using the *R*-packages *sjPlot* and *DHARMa* [24, 25] and tested for statistical significance using the function *Anova* in the *R*-package *car* [26]. We explored pairwise comparisons between generations within each range using the *R*-package *emmeans* [27].

## Results

Parasitism rate was dependent on the interaction between host generation and host ranges (χ^2^_1_ = 5.30, P = 0.021; Fig 1 and Table 1). The parasitism rate in the first generation of *T*. *ekebladella* was higher than in the second generation within the univoltine range (estimate = -1.62, SE = 0.52, P = 0.005; Fig 1, Tables 1 and 2). The parasitism rate did not differ between the first and second generations within the bivoltine range (estimate = -0.11, SE = 0.44, P = 0.806; Fig 1, Tables 1 and 2).

**Table 1:** The effect of host generation, host range and their interaction on the probability of parasitism of the specialist leaf miner *Tischeria ekebladella*. Shown are χ^2^ values, degrees of freedom (df) and P-values of fixed effects, as well as estimates from the variance components, from a generalized linear mixed effects model. Significant P-values (P < 0.05) are in bold.

Response variable	Predictor	χ^2^	df	P
Parasitism rate	Generation	5.10	1	**0.024**
	Range	1.30	1	0.255
	Generation × Range	5.30	1	**0.021**
**Variance components**				
PlantID = 19.51				
LeafID < 0.01				

**Table 2:** *A priori* comparisons of the parasitism rate between the first and the second herbivore generations, separately for the univoltine and bivoltine ranges. Shown are the groups compared, estimates, standard errors (SE), z-ratios and P-values. Significant P-values (P < 0.05) are shown in bold.

Comparison	Estimate	SE	z-ratio	P
First vs. second generation (univoltine range)	-1.62	0.52	-2.82	**0.005**
First vs. second generation (bivoltine range)	-0.11	0.44	-0.25	0.806

## Discussion

In line with our expectations, the parasitism rate of *T*. *ekebladella* was lower in the second than the first generation in the naturally univoltine range, while there was no difference in parasitism rate between generations in the bivoltine range. This pattern suggests that when a new herbivore generation emerges in a previously univoltine population, for instance due to a warmer climate, the existing parasitoid community may not yet be adapted to parasitize this new generation [6, 7]. This may then partly set free the herbivore population from an important mortality factor, resulting in a higher population growth rate.

The fact that parasitism rates were similar for the first and second generations in the bivoltine range might indicate that the lower parasitism rate of the new generation in the univoltine range could be part of a transient stage. In other words, there might be a number of generations after the host has increased in voltinism and before the parasitoid community has caught up. This would be analogous to recent studies that showed that parasitoid communities require some generations to control herbivore populations that have expanded their range [6, 7]. Whether the new generation enters enemy-free space and how long this transient period lasts will depend on the level of specialization of the parasitoids and the mechanisms that synchronize host-parasitoid interactions. In particular, we postulate that herbivores are less likely to enter enemy-free space when the parasitoids are generalists, as generalists are more likely to already attack alternative hosts that are present during the period when the new herbivore generation appears. In contrast, herbivores are more likely to enter enemy-free space when the parasitoids are specialists. An increase in voltinism by specialist parasitoids before an increase by their hosts will be an ecological and evolutionary dead end [5–7, 10]. This scenario is exemplified by our study, where the parasitoids are specialists, and there is low quantitative overlap in the parasitoid community with other leaf miners in the surroundings [16, 17, 28]. We would thus expect at least a transient (if not permanent) period with lower parasitism rate. As for the entry into enemy-free space, the length of a putative transient period will depend on the mechanisms behind the host-parasitoid synchrony. When the parasitoids are generalists, they might quickly catch up with the host. Generalist parasitoids have a high capacity to establish in areas where they were absent when their host expanded its range [5, 10]. While it might take more time, specialist parasitoids might catch up due to intraspecific genetic variation in their response to climate change, allowing natural selection to re-synchronize hosts and parasitoids. Moreover, further increases in temperature might also result in concordant increases in voltinism in the parasitoid due to phenotypic plasticity [5, 11]. It is also possible that parasitoids are constrained in their phenotypic plasticity and evolution, and other natural enemies will start to attack the new herbivore generation.

Our findings raise many new fundamental and applied questions, several of which are particularly urgent to address given the increase in voltinism with climate change. A couple of important questions to answer are how long the transient stage with a higher parasitism rate in the new generation will last and whether the temporarily lower parasitism rate in the new generation will translate into higher herbivore infestation levels, higher plant damage and lower plant population growth rates. Another important direction for future research, we propose to carry out field experiments with multiple host species and in other systems to explore the generality of our findings. While beyond the scope of our study, our findings suggest that the parasitism rate of the first generation is higher in the univoltine range than in the bivoltine range (Fig 1, Table 3). While geographic differences in average parasitism rates can be caused by many factors, such as spatial variation in temperature, humidity, parasitoid community structure and virulence traits, as well as stochasticity, it would be interesting to know whether persistent differences in parasitism rate exist between univoltine and bivoltine ranges [5, 10, 11, 29].

**Table 3:** *A posteriori* comparisons of the parasitism rates between the univoltine and bivoltine ranges, separately for each herbivore generation. Shown are the groups compared, estimates, standard errors (SE), z-ratios and P-values. Significant P-values (P < 0.05) are shown in bold.

Comparison	Estimate	SE	z-ratio	P
Univoltine vs. bivoltine range (first generation)	0.95	0.41	2.31	**0.021**
Univoltine vs. bivoltine range (second generation)	-0.62	0.55	-1.13	0.260

Finally, while our study took the perspective of the herbivore, future observational and experimental field studies might explore differences in the structure of the parasitoid community and their level of specialization between herbivore generations. Mechanistic studies might target the underlying physiological mechanisms by which specialist and generalist parasitoids track, and adapt to, their hosts. For example, the development of specialist parasitoids might in some instances be physiologically linked to the host (e.g. due to hormones produced in the host while the parasitoids are developing). In such cases, parasitoids can instantaneously catch up with changes in voltinism in the host.

### Conclusions

Our findings provide the first evidence that herbivores may enter enemy-free space through climate-induced changes in host voltinism. As climate-induced changes in host voltinism are frequently observed, our study might characterize a common but thus far understudied phenomenon. We provide explicit hypotheses on how to generalize the concept of voltinism-related enemy-free space to other species and systems by considering the degree of specialization of the parasitoid community and the underlying mechanisms of host-parasitoid synchrony. Taken together, our study contributes to our understanding of the effects of climate change and shifts in voltinism for plant-based species interactions and generates novel questions and hypotheses that are important and timely for future research.
